# Supplementary material for: A super-pangenome of the North American wild grape species
Source: Genome Biol. 2023 Dec 19;24:290. doi: 10.1186/s13059-023-03133-2 (PMC10729490; doi:10.1186/s13059-023-03133-2)
Supplement: Supplementary file 2 — Additional file 2: Figure S1. Frequency of variants between the haplotypes of each genome after intra-genomic comparisons. Figure S2. Graph-based pangenome modeling. Figure S3. Transposable elements composition in the pangenome. Figure S4. Comparison of the final pangenome sizes. Figure S5. NBS-LRR gene distribution. Figure S6. Frequency of the variant type modifier in the different pangenome classes. Figure S7. Structural variants at the sex-determining region within the VviINP1 gene. Figure S8. Consistency of the detected variants in the pangenome graph with the previously PDR-associated regions. Figure S9. Single-reference GWAS and root chloride association analysis. Figure S10. PGGB modules optimization. [file 13059_2023_3133_MOESM2_ESM.pdf]

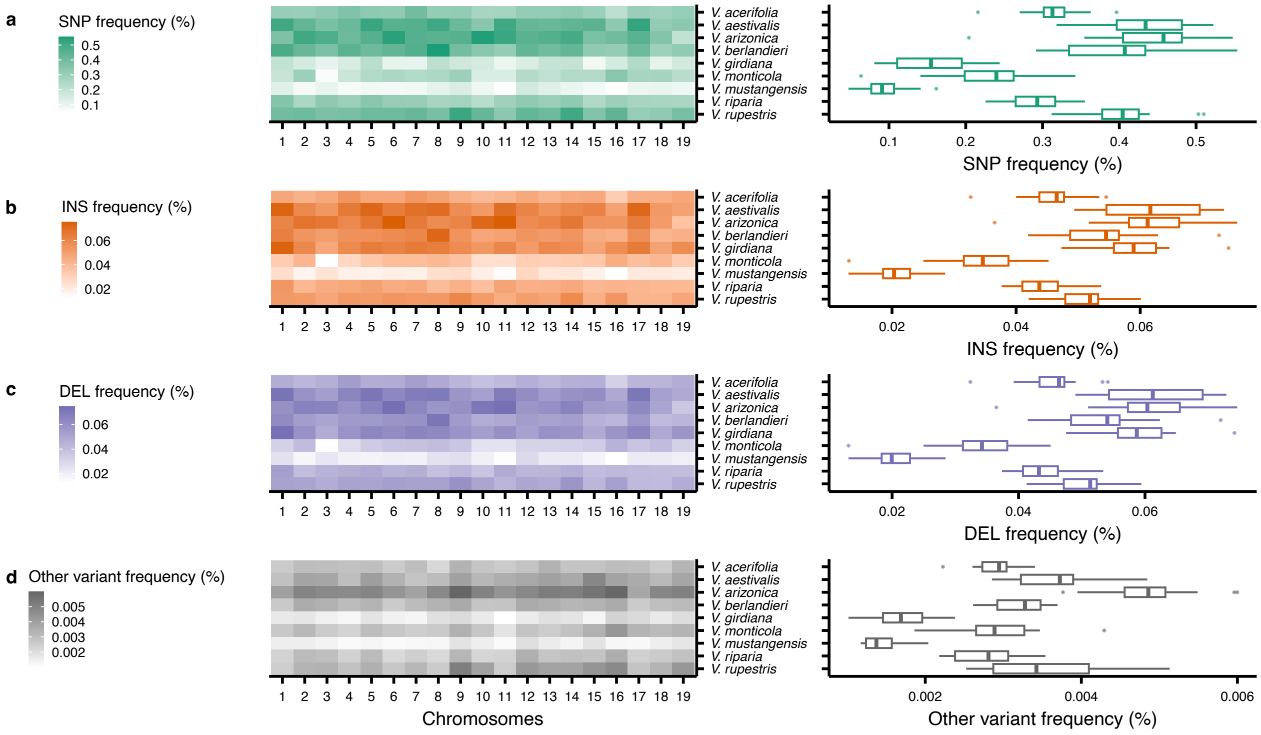

Figure S1. Frequency of variants between the haplotypes of each genome after intra-genomic comparisons. The heatmaps on the left side represent the number of variants per base for each chromosome as a percentage of the total chromosome length. On the right side, the boxplots summarize the same information at genome level. The middle bars represent the median while the bottom and top of each box represent the 25th and 75th percentiles, respectively. The whiskers extend to 1.5 times the interquartile range and data beyond the end of the whiskers are plotted individually as outlying points.

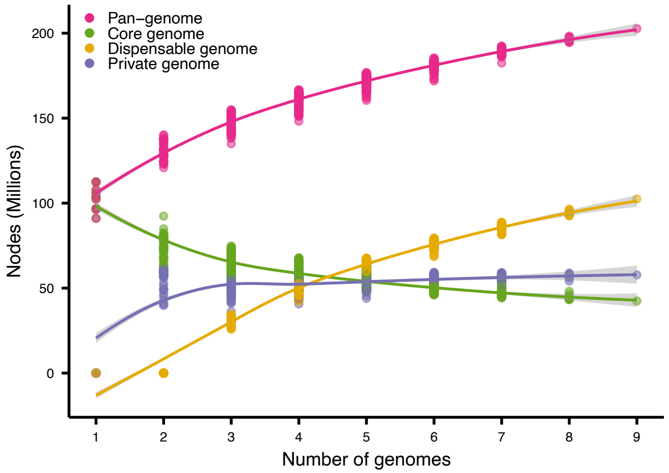

Figure S2. Graph-based pangenome modeling. For every combination of 1-9 genomes, the total number of the unique nodes is represented per class. The lines represent smoothed conditional means with a 0.95 confidence interval.

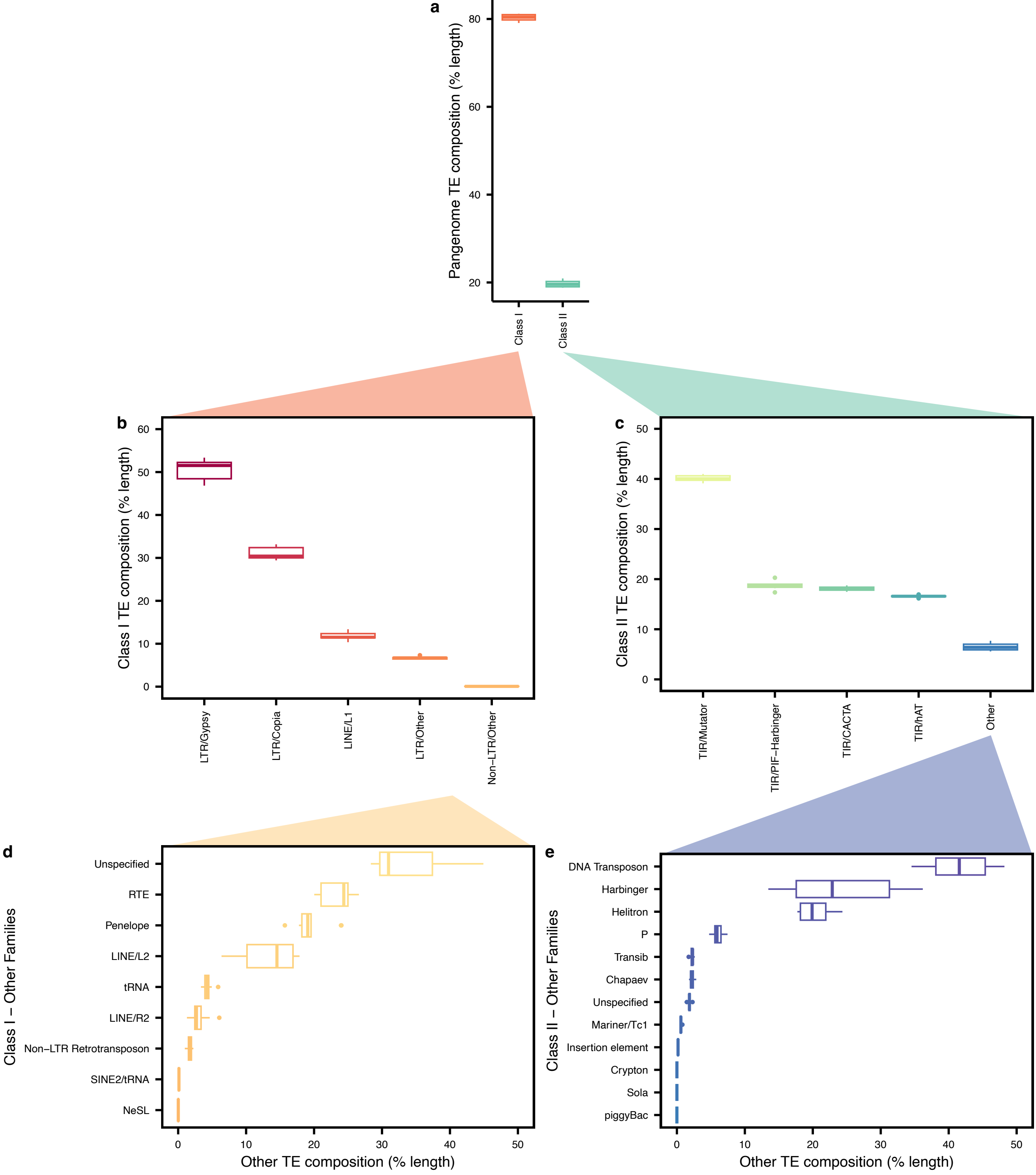

Figure S3. Transposable elements composition in the pangenome. The TEs are divided into two main classes (**a**), subdivided in subclasses (**b**, for Class I; **c**, for Class II). The content of the subclass "Other" is detailed for Class I (**d**) and Class II (**e**). The middle bars represent the median while the bottom and top of each box represent the 25th and 75th percentiles, respectively. The whiskers extend to 1.5 times the interquartile range and data beyond the end of the whiskers are plotted individually as outlying points.

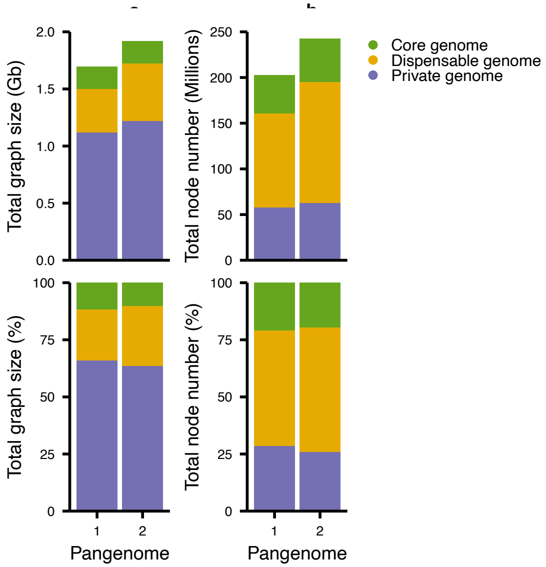

Figure S4. Comparison of the final pangenome sizes. The pangenome 1 was built using the nine distinct parental *Vitis* species while pangenome 2 contains the additional genomes of three hybrids. The graph size and the node number are represented as absolute numbers per genome class in **a** and **b**, respectively, and in percentage in **c** and **d**, respectively.

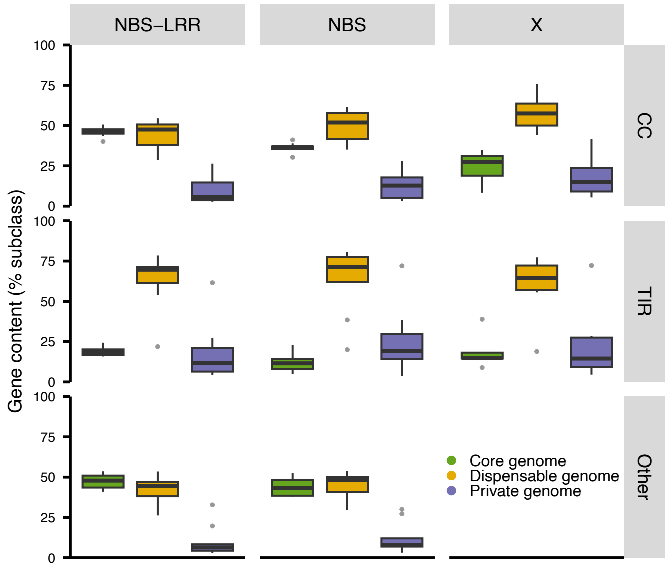

Figure S5. NBS-LRR gene distribution. For each main class of NBS-LRR represented in rows, the distribution of the gene content is represented per class of genome in the pangenome (e.g. the top-left plot represents the CC-NBS-LRR genes). The middle bars represent the median while the bottom and top of each box represent the 25th and 75th percentiles, respectively. The whiskers extend to 1.5 times the interquartile range and data beyond the end of the whiskers are plotted individually as outlying points.

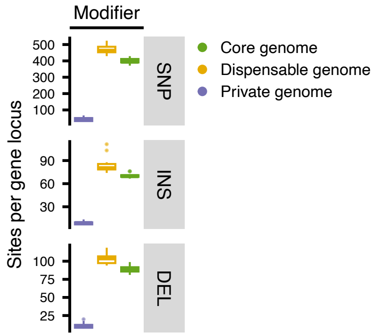

Figure S6. Frequency of the variant type modifier in the different pangenome classes. For each variant type, the number of sites annotated as modifier is represented (n = 18 haplotypes). Each genome class has a distinct color. The middle bars represent the median while the bottom and top of each box represent the 25th and 75th percentiles, respectively. The whiskers extend to 1.5 times the interquartile range and data beyond the end of the whiskers are plotted individually as outlying points.

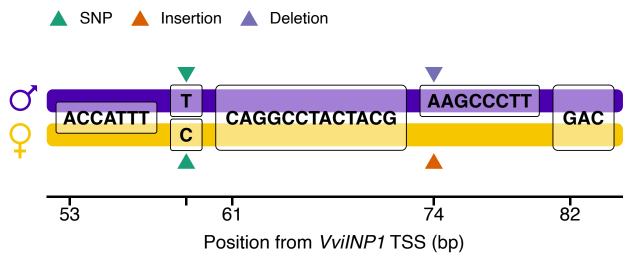

Figure S7. Structural variants at the sex-determining region within the *VviINP1* gene.
The x-axis represents the position from the transcriptional start site (TSS) of *VviINP1*. The two horizontal bars represent the alleles of the male (purple) and the female (yellow) *VviINP1* coding sequence. Common nucleotides are presented in the middle of the two bars while allele-specific bases are indicated on the corresponding horizontal bar. Variants are indicated as triangles, SNPs in the two references are colored in green, the deletion in the male (when compared with female) is in purple, and the insertion in the female (when compared with male) is in orange.

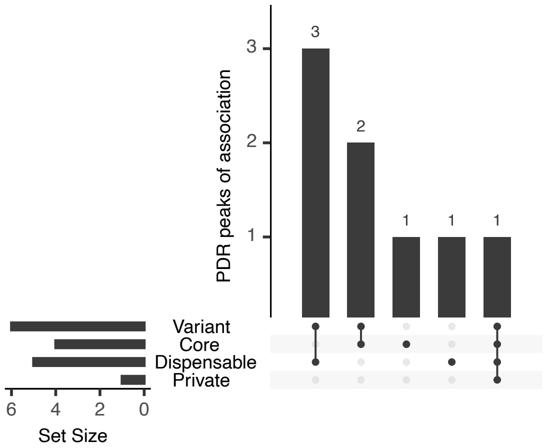

Figure S8. Consistency of the detected variants in the pangenome graph with the previously PDR-associated regions. The vertical bars represent the number of PDR-associated regions detected or not as variants in the pangenome graph (first row) and how the corresponding nodes were classed in the pangenome (core, dispensable, private). If a line connects two dots, it means that the nodes belong to these two types of classification, e.g., the nodes corresponding to the three first PDR-associated peaks were classed as variants and belong to the dispensable genome. The set size illustrated by horizontal bars is another representation of the total number of peaks belonging to the different classes indicated in rows (variant, core, dispensable, private).

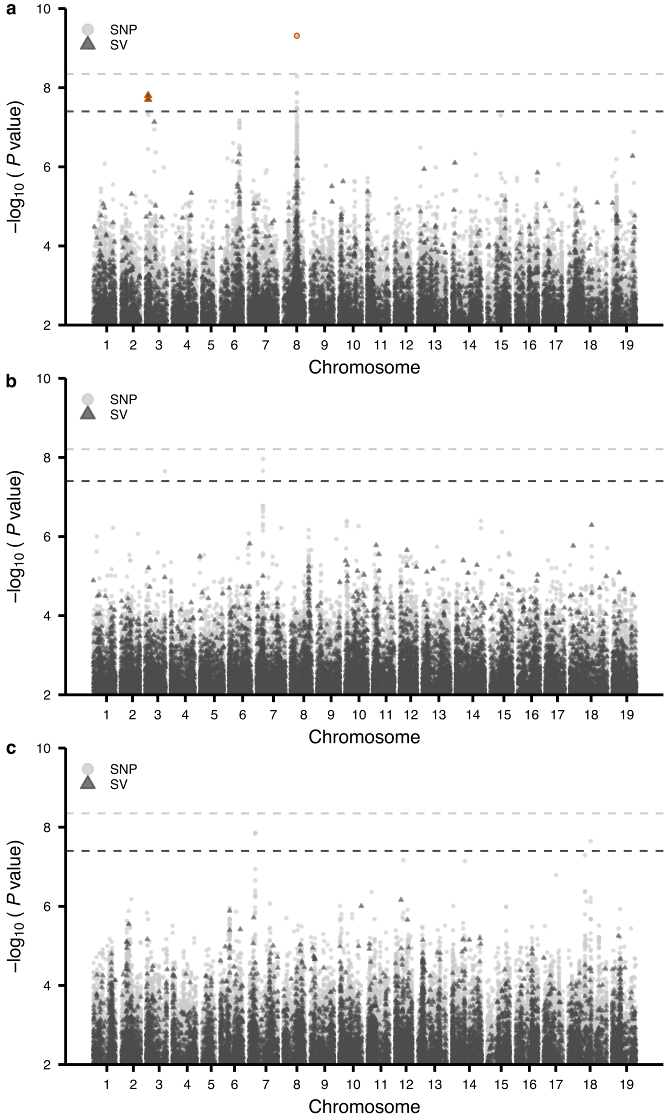

Figure S9. Single-reference GWAS and root chloride association analysis. a, Manhattan plot representing significant association with the chloride concentration in the leaves after the single-reference GWAS. **b** and **c** represent the results for association analyses performed with the chloride concentration in the roots from the pan-GWAS and the single-reference GWAS, respectively. Significant associations are detected using a Bonferroni threshold set to -log10(0.05/n) and colored in orange. The SNPs are represented as light grey points, the SVs as dark grey triangles.

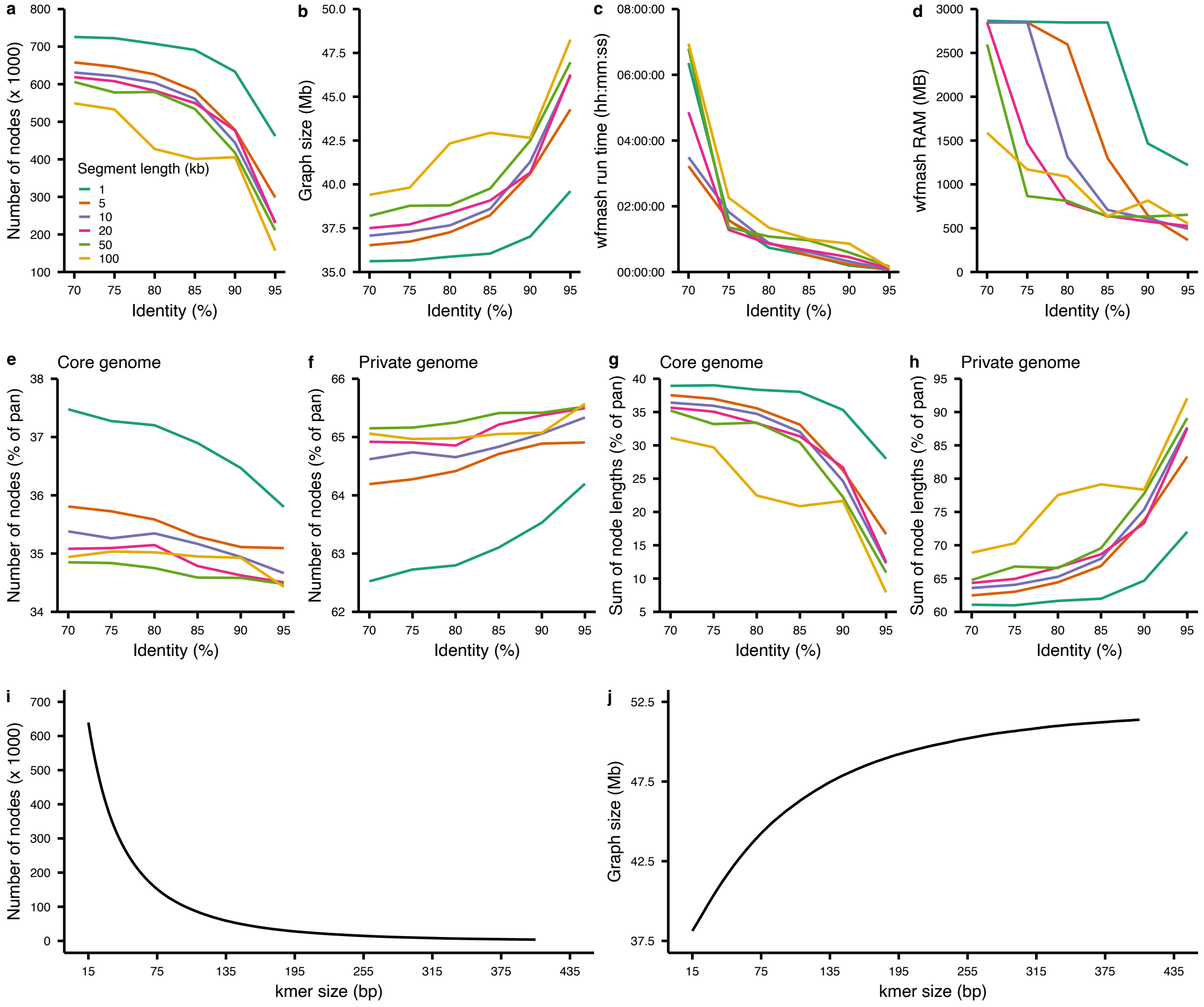

Figure S10. PGGB modules optimization. For wfmash, the segment length (1, 5, 10, 20, 50, and 100 kb) and the percentage of identity (70, 75, 80, 85, 90, 95) parameters were tested while seqwish was ran afterwards with a fixed kmer = 19 bp. For each constructed graph, the number of nodes (**a**), the graph size (**b**), the run time (**c**) and RAM required by wfmash (**d**), the number of nodes in the core (**e**) and private (**f**) genomes and their total lengths (**g** and **h**) relative to the pan-genome are represented. With a fixed segment length of 10 kb and a percentage identity of 85%, the number of nodes (**i**) and the size of seqwish graphs (**j**) were evaluated with kmer sizes ranging from 15 to 405 bp, every 2 bp.
