# Supplementary material for: A super-pangenome of the North American wild grape species
Source: Genome Biol. 2023 Dec 19;24:290. doi: 10.1186/s13059-023-03133-2 (PMC10729490; doi:10.1186/s13059-023-03133-2)
Supplement: Supplementary file 3 — Additional file 3. Review history. [file 13059_2023_3133_MOESM3_ESM.docx]

Review History

**First round of review**

**Reviewer 1**

**Were you able to assess all statistics in the manuscript, including the appropriateness of statistical tests used?**

Yes, and I have assessed the statistics in my report.

**Comments to author:**

In this manuscript, the authors reported the haplotype-resolved genome assemblies of the representative accession of nine species in Vitis genus. Based on the pairwise whole-genome alignments of these genomes, a reference-unbiased super-pangenome was constructed, and thus their comprehensive genomic diversity was revealed. Moreover, the authors attempted to identify the variants conferring the adaptive traits such as salt tolerance. Overall, these genome resource and detected variants will be an important resource for community. However, more data, experiments and indepth analysis are required to ensure the high-quality genome and provide solid novel insights into the biology of grape.

Specific comments:

1. The authors claimed that they constructed a super-pangenome for Vitis genus using the representative genomes of nine species. Do the nine species represent nearly full spectrum of the Vitis genus? From Figure 2b and Figure 3a, it seems that these genomes could not be genuinely enough to represent the super-pangenome. I would expect a "diminishing return" experiment showing that as more and more genetically diverse genomes are added to the pipeline then progressively fewer and fewer dispensable sequences and genes are found. I suggest that the authors do not mention super-pangenome, and just focus on the newly assembled genomes and identified variants.

2. In the title, the authors overstate the findings about genus-wide association with adaptive traits. In this MS, however, only pan-GWAS was performed for salt tolerance using 153 samples from 12 species.

3. Accurate phasing of the two haplotypes is challenging for the heterozygous diploid genome. Here, the diploid sets of chromosome sequences were reconstructed using Haplosyn and a high-density Vitis genetic map. It is important to provide additional validation for phasing accuracy. For instance, the authors can utilize the Hi-C interaction heatmap of the diploid genome or validate the haplotype correctness using the published complete sequences of long DNA fragment.

4. In the section "The nucleotide-level super-pangenome graph of the North American Vitis genus", they found that 27.10 to 34.20% of the genome from each species was affected by genetic variants. I wonder whether some of them are derived from the incorrect haplotyped sequences.

5. The characterization of variants between the two haplotypes of each genome was insufficient. With the high-quality phased genomes, these can be comprehensively analyzed, and some novel insights may be offered.

6. Three hybrid varieties derived from the cross of the species included in the nine genomes were incorporated into the super-pangenome to assess their origins and genetic effects of different genomic components. However, no apparent novel findings were provided in this version. More indepth analysis are expected to provide interesting results.

7. Pan-GWAS was conducted to detect significantly associated variants with salt tolerance. On chromosome 8, a significant peak was observed, but the causative variants were not proposed. The upregulated expression under salt treatment and the known function of homolog in other crops were not sufficient to support it as the candidate gene conferring salt tolerance.

**Reviewer 2**

**Were you able to assess all statistics in the manuscript, including the appropriateness of statistical tests used?**

No, I do not feel adequately qualified to assess the statistics.

**Comments to author:**

Cochetel et al. 2023 constructed a super-pangenome incorporating 9 north American wild grapevine genomes (haploid resolved) to investigate genomic variation across these individuals. They report intra- and intergenomic variation and test their super-genome by trying to detect variation previously reported for hybrids, sex determination, PD, and salt tolerance. Wild grapevines are an important resource of genetic diversity and understanding the genetic variation across genera is an important resource for grapevine improvement. Looking at wild grapevine genetic diversity at this scale is an advancement from previous wild grapevine studies. There are some questions and comments below that will help strengthen this manuscript:

General comments:

-Connect your super-pangenome to use in other species for a broader impact of your work.

-Pangenomes are made up of multiple genomes of the same species to detect genetic diversity. Here, you only use one diploid genome (2 haploid) to construct the super-pangenome. I think a lot of intra-species variation is lost. The name "super-pangenome" is a little misleading, since pangenome implies multiple species' genomes are included. I think species-level pangenomes need to be assembled first, then they can be used to generate a genus (super) pangenome. This needs to be addressed in the manuscript. Otherwise, a different name might be more appropriate than "super-pangenome".

Comments by line:

Line 66- 86: You mention a single reference genome affects the intraspecific genetic variation, missing much of the genetic novelty within species, but then your super-pangenome only uses one genome per species.

Line 70: genetic landscape "within a species" that may…

Line 103: When you say "respective groups" do you mean species or are you talking about the 6 groups from the PCA analysis in Fig 1j? Please clarify why/how these were selected.

Line 108: 791-6,925?

Line 110: Hybrid assemblies of both haplotypes?

Table 1: In the "Repeats" column, is this the % of the assembly that consisted of repetitive elements?

Table 1 footnote: Delete the first sentence and just keep the second. It's not needed with he super script ones already in the table.

Line 123: Re-word. and "were used to constrict" a high-density Vitis…

Line 131: These heterozygosity values for the wild grapevine genomes are high compared to what was previously reported. What was included in this calculation?

Line 144: Fig S1 doesn't really add any new insights into the phylogenetic relationships compared to Fig. 1k, so it might not be necessary to include. Or you can include more of an explanation of why it is.

Line 144: What do you mean by "clock calibration"? Cite a source or include more of a description here.

Line 145-147: Are the 450 rapidly evolving orthogroups specifically pictures in Fig. 1k? If so, highlight them/make them more apparent. If not, remove the figure citation.

Line 154: delete "from"

Line 156: include the heterozygosity values again here in parentheses for the reader.

Line 167: You use the term "node" often, but never define what these are. Please define this the first time you reference it.

Line 171: replace "species-specific" with "genome-specific" since you only have one representative genome per species.

Lines 193-194: Clarify the meaning of this sentence, particularly "their effect on genetic components".

Line 202: Were the core and private genomes used the ones already determined by the super-pangenome or were these generated again for the three species and their hybrids compared here? Please clarify.

Line 205: 57.2% of what? Also, I am not seeing this pictured in Fig S5a. In the text you say 57.2% were shared between the two haplotypes. This means about 43% (of the sequences?) were unique to the haplotype? That seems high.

Line 212: Replace "in" with "on".

Line 213: What % of the hybrid genomes was the parental genomes able to explain. All of it?

Line 220: Significant time and space is spent on this hybrid comparison, so would be better to have a main figure to illustrate your findings.

Line 229: Discarded for or excluded from? Were they set aside to study specifically?

Line 229: What did you find related to the other categories. It seems like this is what you set out to find, but you don't report on it in the text or main figures. The % of core, dispensable, and private genes? The reader expects a discussion on this, but this comes later. You can re-word or restructure this paragraph so it has more of a logical flow.

Lines 247-249: If the genes arose through duplication, TEs could also be a mechanism for origination.

Line 254: Do you mean genes in these rapidly evolving gene families? Fig 3K looks to show % of genes.

Line 261: This is interesting, since, at this level of GO, it looks like some of these terms are related to basic functions, like embryo development. Maybe some of these variable genes evolved important/essential functions in their species/individual.

Line 267: I don't think you can make this conclusion without functional tests. Maybe a more general statement can be made.

Line 277: delete "across"

Line 278: change 86 to 76%? Check table S16.

Line 281: re-word

Line 281: delete "most"

Lines 286-287: Put "except for the multiple nucleotide polymorphisms (MNP)" outside of the parentheses and then site S7a.

Line 294: Is this because SV was not able to be detected outside of the haplotypes of the species? What is a modifier site?

Line 298: It's worth to briefly mention how this gene contributes to sex determination.

Line 302: Any new or genome-specific variation of interest in the SDR discovered via the super-pangenome?

Lines 304-311: You found SNP variants at these regions, and you would expect to see this, since V. arizonica is included and had those SNPs. Were you just verifying if you could pick up on these SNPs in the pan genome or were you trying to see if you could predict PD resistance of other species included in the analysis by determining if they also had them? Are these known conserved genes involved PD across species? Do you know the PD resistant phenotype of the other species? Can you take this analysis a step further and make predictions? Clarifying the aims of this analysis will make it clearer for the reader.

Line 312: Mapping the reads or do you mean identifying SNPs/variation from the last GWAS?

Line 355: Can you determine if it is an expression difference or maybe a gene dosage difference contributing to the salt tolerance?

Line 386: And can influence gene duplication via unequal crossing over. You can elaborate a little more about TEs in the discussion and role in genome evolution.

Lines 392-393: Why?

Lines 417-419: This should be mentioned in the results section and the discussion of the role of these genes in other species can be discussed here.

Lines 426-441: This is somewhat repetitive with the discussion.

Line 445: List the tissues or say (see corresponding sections below).

Line 448: maintained in the field or greenhouse

Line 448: Was there a specific reason you selected the genera you did? Availability of genome sequences, plant material, or phylogenetic relationships? State this here.

Line 462: Delete "a"

Line 503: Specify if the sequences were assembled into consensus contigs or haplotype-resolved.

Line 515: BUSCO scores

Line 523: Align haplotype of each genome against each other or against the primary haplotype?

Lines 523-524: Variants of any size? More detail is needed here, specially since your heterozygosity scores are higher than past reports for wild grapevines.

Lines 591-595: Again, please define what a node id and sequence are comprised of. Are these sequences of a defined length, coding and/or non-coding, single nucleotides?

Line 614: define "decoys"

Main Figures:

Fig 1- include the name of the species in panels a-i or somehow connect species names in j and K with the species in panels a-i. Also, these species are dioecious, right? Indicate if the flowers/individuals pictures are male or female.

Fig 3g: The bar graph looks off. Are the white boxes supposed to show the % of the genes without annotated domains? It's better to make the outlines of the bars black if that is the case.

Fig 3k- change Y axis label to % genes in expanded/contacted gene families

Fig 3l, m: would be helpful to add light lines from the category to the bubbles, especially for the core genes, so it is easier to see which one corresponds to which.

Fig 4a- define abbreviations in legend.

Fig 4d- The Y-axis descriptions are not clear from the figure or the legend. Why is intron listed in the top row and bottom row? Are they referring to different types of mutations in the intron? Why is "disruptive" and "conservative inframe" listed with "insertion" and "deletion" after it, when those are headings of the columns? Is "high", "moderate" and "low" referring to synonymous, missense, and stop codons or to the impact of all types of mutations based on location within the gene? Please clarify.

Figure 4f: Fig 4f is difficult to interpret given the current legend. It is not clear what the dots and lines connecting them mean. Why are the 8 peaks separated as they are in the vertical bar graph. Does "set size" mean number of genomes that had SNPs? Please clarify.

Fig 5a- Are there supposed to be lines connecting the boxes? It looks like there is a lot of white space in this panel.

Supplements:

Sup Table 1- Write out FPS abbreviation

Sup Table 2- Why is the N50 for B40-14 so low compared to the others? Check this value.

Sup Table S3- Why does V. riparia not have values in this table. Include a note under the table or in the M&M to explain.

Sup Table 4- Why no Iso-seq for V. rupestris?

Sup Table 9- define SD. Did 9031, HP-1 and B38 not have genome size predictions?

Sup Table 14- define/rename "number" and "total" so it is more descriptive. Is one for graph inferred and the other orthology?

Sup Table 16- What is length and coverage referring to? The title says consistence statistics between nucmer and vg deconstruct but that comparison is not clear from the headers.

Fig S5a and b: In S5a, if this is an intragenomic comparison, then why is the X-axis the pangenome length? Does incorporate all the data for the 3 species? Isn't this showing what percentage of the haplotypes that consisted of core and private sequences? There is a disconnect between what is discussed in the manuscript and what is shown in Fig S5. A more descriptive legend or a more detailed description would help connect the figure to the manuscript text. In S5b, label your samples better and/or tell what the IDs mean in the legend. Also, I don't see an "outline color" that is supposed to denote the genome class. What do the yellow, orange, red, purple, blue, and teal colors mean? Do they specify a specific genome? This needs a key.

Sup Tables in general- Mention why data points are missing in supplemental tables for specific genomes.

**Authors Response**

**Point-by-point responses to the reviewers’ comments:**

Reviewer #1:

In this manuscript, the authors reported the haplotype-resolved genome assemblies of the representative accession of nine species in Vitis genus. Based on the pairwise whole-genome alignments of these genomes, a reference-unbiased super-pangenome was constructed, and thus their comprehensive genomic diversity was revealed. Moreover, the authors attempted to identify the variants conferring the adaptive traits such as salt tolerance. Overall, these genome resource and detected variants will be an important resource for community. However, more data, experiments and indepth analysis are required to ensure the high-quality genome and provide solid novel insights into the biology of grape.

Specific comments:

1. The authors claimed that they constructed a super-pangenome for Vitis genus using the representative genomes of nine species. Do the nine species represent nearly full spectrum of the Vitis genus? From Figure 2b and Figure 3a, it seems that these genomes could not be genuinely enough to represent the super-pangenome. I would expect a "diminishing return" experiment showing that as more and more genetically diverse genomes are added to the pipeline then progressively fewer and fewer dispensable sequences and genes are found. I suggest that the authors do not mention super-pangenome, and just focus on the newly assembled genomes and identified variants.

*Reply: Thank you for the suggestion. We revised the manuscript carefully and made sure to tone down or remove any statement that could imply that the pangenome we produced is meant to represent the full Vitis genus. We made sure we never use “The” and instead use “a” to describe the pangenome and clearly state that it only refers to species from North America as specified in the manuscript title. While the pangenome size is shown to continuously increase after adding nine genomes, we believe that it can still be considered as a super-pangenome. According to the consensus definition from the literature, a super-pangenome is a pangenome including multiple species. Published examples further illustrate such definition with plant super-pangenomes represented by multiple species such as in watermelon (3 species used) or in tomato (11 species selected). Based on the current literature consensus, we believe that defining the pangenome we built as a super-pangenome stands correct.*

2. In the title, the authors overstate the findings about genus-wide association with adaptive traits. In this MS, however, only pan-GWAS was performed for salt tolerance using 153 samples from 12 species.

*Reply: We agree with the reviewer. We replaced adaptive traits by salt tolerance in the title as suggested.*

3. Accurate phasing of the two haplotypes is challenging for the heterozygous diploid genome. Here, the diploid sets of chromosome sequences were reconstructed using Haplosyn and a high-density Vitis genetic map. It is important to provide additional validation for phasing accuracy. For instance, the authors can utilize the Hi-C interaction heatmap of the diploid genome or validate the haplotype correctness using the published complete sequences of long DNA fragment.

*Reply: In place of Hi-C, genomes were constructed using BioNano for optical mapping, which in our experience facilitate phasing while improving contiguity of the pacbio contigs. In our experience, in heterozygous genomes such as grapes, Hi-C scaffolding tends to merge haplotypes which then require extensive manual deconvolution. This issue is one of the numerous reasons we developed the tool haplosync (Minio et al., 2022 G3 12(8), jkac143). Haplosync uses external information to associate sequences to chromosomes, in our case we used a high-density genetic map that contains markers conserved in the Vitis genus. Markers and assembly information (relation primary contigs/haplotigs in our case) are used as constraints to simultaneously phase the sequences into two haplotypes and attribute them to a chromosome. Any potential conflict during the scaffolding is resolved manually using interactive visualizations. The phasing correctness is assessed after each step of the scaffolding process by testing for gene duplication and DNA-seq coverage. During the last steps, the remaining unplaced sequences are used to fill the potential gaps left in the assemblies using the pairwise relationship between the two haplotypes ensuring the completeness of each haplotype. We added more details L124-132 regarding the tool and how it provides an accurate phasing. We also highlighted the BUSCO scores per haplotype in the text L149-150 to add more emphasis regarding the haplotype completeness.*

*Lines 124-132: “The tool HaploSync [12] coupled with a high-density Vitis genetic map [13] was used to construct nine diploid sets of nineteen chromosomes with a target of at least 90% of anchored sequences and protein-coding gene loci (Table 1). For each genome, the markers and the relation primary contigs/haplotigs were used as constraints to simultaneously phase the sequences into two haplotypes and attribute them to a chromosome. The phasing correctness was evaluated at every step of the scaffolding using both the allelic gene content and DNA sequencing (DNA-Seq) short-read coverage (Additional File 1: Table S5) to ensure the accuracy of the diploid phasing and prevent scaffolding issues such as haplotype switches. Gap filling was performed with the remaining unplaced sequences based on the pairwise relationship between the haplotypes.”*

*Lines 149-150: “The phasing integrity was further supported by the BUSCO scores at 93.2 2.0% for haplotype 1 and 89.2 4.7% for haplotype 2 (Table 1).”*

4. In the section "The nucleotide-level super-pangenome graph of the North American Vitis genus", they found that 27.10 to 34.20% of the genome from each species was affected by genetic variants. I wonder whether some of them are derived from the incorrect haplotyped sequences.

*Reply: The pairwise comparisons we performed here contrast the different species and do not include any intra-species comparisons. In the scenario of a haplotype switch, our estimations should remain very similar or identical as the percentages average the values from the two haplotypes. We added the term “between species” L171-173 to make it clearer.*

*Lines 171-173: “After pairwise comparisons of assemblies between species, we inferred that 27.10 to 34.20% of the genome from each species was affected by genetic variants (Additional File 1: Table S11), values that greatly exceeded heterozygosity (16.79-23.77%).”*

5. The characterization of variants between the two haplotypes of each genome was insufficient. With the high-quality phased genomes, these can be comprehensively analyzed, and some novel insights may be offered.

*Reply: While comparing haplotypes within species was not the focus of the paper, we agree with the reviewer that more insights could be obtained and performed several additional analyses:*

*1) Independently from the pangenome and relying solely on the pairwise comparisons of haplotypes withing each genome (nucmer), we now present the distribution of the variant frequency along the chromosomes L143-146 and illustrated on the Additional File 2: Figure S1.*

*Lines 143-146: “SNP was the most abundant type of variant with 1 SNP every 238-1159 bp (Additional File 2: Figure S1) as reported previously for wild grapes species [16,17]. INDELs were more spread with an average distribution six times wider than for the SNPs (1 INDEL every 1652-5030 bp) (Additional File 2: Figure S1).”*

*2) The pangenome graph was also used to infer the variants between haplotypes of every single species and we provide SNP frequencies L312-315 supported by Additional File 2: Table S17.*

*Lines 312-315: “For each species, 1 heterozygous SNP was detected every 239-888 bp between haplotypes representing 0.11-0.42% of their assembly (Additional File 1: Table S17) which is consistent with our independent pairwise comparison using NUCmer and reports from literature [16,17].”*

*3) After extracting the variants, we analyzed their impact on gene function with an emphasis on hemizygous genes. The results are presented L332-335 and Fig. 5d.*

Lines 332-335: “Within the genes impacted by the variants, we decided to focus on the hemizygous gene sets we previously identified. We observed an overall higher abundance of variants in these gene loci (Fig. 5d) with more than 2x more sites on average for SNPs with moderate impact.”

*4) We performed an enrichment analysis on the variant effects for the hemizygous genes L335-337 presented on Fig. 5e.*

*Lines 335-337: “Among the effects with high impact, transcript ablation and exon loss variant were the most enriched in the hemizygous gene loci compared with the total content of genes impacted by variants (Fig. 5e).”*

*In summary, now the manuscript contains a comprehensive characterization of the variants between the haplotypes of each genome. The NUCmer gives a great overview of the variant distribution along the 19 chromosomes of each genome and allows also to perform comparisons between genomes. As these values were previously used in literature to assess heterozygosity in wild grape species, we can directly compare our results to previous reports. The second analysis using the variants from the pangenome graph decomposition provides an independent support to the pairwise comparisons between haplotypes and reinforces the accuracy of the variability captured by the pangenome graph. We also characterized the variants associated with the presence of hemizygous genes. While hemizygosity was estimated independently of the pangenome, the graph showed significant enrichments of variant impacts and effects that are detrimental to the presence of these genes in one of the two haplotypes. Altogether, we believe that our new analyses comparing the structure, the pangenome topology and the gene space between the haplotypes of each genome represent a great addition to the manuscript and provide a comprehensive overview of the intra-genomic variability.*

6. Three hybrid varieties derived from the cross of the species included in the nine genomes were incorporated into the super-pangenome to assess their origins and genetic effects of different genomic components. However, no apparent novel findings were provided in this version. More indepth analysis are expected to provide interesting results.

*Reply: We re-analyzed the data and reshaped entirely the figure now presented as Fig. 3. We simplified how similarity is estimated between haplotypes now represented as a shared node space. Briefly, for every comparison of interest (indicated as a black line between haplotypes on Fig. 3), we extracted the nodes for the haplotypes compared. Their frequency was calculated to determine whether they were shared or present only in one of the two haplotypes compared. The shared nodes were represented as a shared node space by comparing the total length of the shared nodes to the total length of the nodes from the haplotypes compared. In addition of the chord diagrams that depict the single comparison, we also present different data summaries to highlight important insights. The first barplot gives a great idea of the haplotypes divergence in hybrids compared with the parental species.*

*Lines 229-233: “Notably, the parents presented a higher percentage of shared node space between their haplotypes (65.9% on average) compared with the hybrids (54.4% on average). The hybrids having V. berlandieri as a parental species showed the lowest percentage (Richter 110R: 51.7%; Kobber 5BB: 50.9%), probably because this species is genetically distant from the two other parents V. riparia and V. rupestris (Fig. 1k).”*

*We believe that this representation fits better to the description we provide in the results section. Furthermore, the comparisons hybrids-parents and between genomes were summarized in boxplots and further confirm the similarities between V. riparia and V. rupestris, result that was not accessed in our previous analysis design.*

*Lines 235-239: “When we compared each hybrid haplotype (e.g. 110R haplotype berlandieri annotated 110R be on Fig. 3d) with the haplotypes of its parental genome (i.e. V. berlandieri haplotype 1 or 2 annotated be hap1 or hap2 on Fig. 3d), we found on average 58.2% of their nodes being shared. V. rupestris showed the most conserved node space with its corresponding hybrids 110R and 101-14 Mgt (Fig. 3e-f).”*

*Lines 241-244: “As expected, V. riparia and V. rupestris haplotypes showed the highest shared node space since there are closer to each other than V. berlandieri. Despite those similarities, the overall shared node content between different species remained lower than the percentages observed when hybrid haplotypes were compared with their parental species.”*

*This whole approach was designed to assess if we can use the pangenome to evaluate the genetic similarities between the hybrid and their parental species and class them in a relative manner from intra-species to inter-specific configuration. We added an explanation L210-214 to make sure it is clear for the reader.*

*Lines 215-219: “We incorporated these hybrid genomes to evaluate whether we could accurately assess their origins and to characterize their structural effects on the pangenome graph topology. We established different types of comparisons to assess whether the pangenome graph was able to reflect correctly different degrees of similarity between close or distant species.”*

*The sole purpose of this analysis is to use more genomes and confirm how accurate remains the overall pangenome structure. The additional genomes are supporting information in this case. Based on our results, we observed that the pangenome correctly reflects the similarities or differences between close and distant species. We attempted other approaches not presented here to provide you with more insights regarding heterosis for example, unfortunately, as the pangenome was not build for this purpose, such analyses require additional data and work which is beyond the scope of the present study. For example, the node space from the parents that is transmitted and conserved in the hybrids was evaluated for the 3 parents to understand if any functional categories or gene functions was enriched when compared with the other parental genomes. However, relying on a shared node space in a subset of species from a larger pangenome does not guarantee the correct representation of the gene space and would actually bias the final outputs. It would require the combination of two information categorizing the genes in pangenome classes as we did before but also based on the parental-progeny relationship by identifying colinear genes between parents and hybrids. We would encounter the same limitations if we tried to study the gene content overall as their classification as core, dispensable, and private would be pangenome wide. In addition, the classification of colinear genes between each pair of hybrids-parents haplotypes and their interconnections in a configuration that contain 3 trios of parents-hybrids represent a significant amount of work and could be another manuscript by itself. The primary objective of this pangenome was not meant to focus on rootstocks as they are only instrumental in supporting the pangenome structure.*

7. Pan-GWAS was conducted to detect significantly associated variants with salt tolerance. On chromosome 8, a significant peak was observed, but the causative variants were not proposed. The upregulated expression under salt treatment and the known function of homolog in other crops were not sufficient to support it as the candidate gene conferring salt tolerance.

*Reply: Thank you for the suggestion. We agree that further experimentation is necessary to determine the role of these genes in salt tolerance. We agree that their role remains hypothetical, but we still think that we can present them as genes of interest for further experimentation. “Candidate” doesn’t imply certainty, but rather suggests potential relevance based on existing evidence, functional conjecture, and statistical association with the trait as in this case. We made sure to tone down any comment implying that we identify a gene that explain salt tolerance and suggest further characterization to validate the function of these genes L497-499.*

*Lines 497-499: “Altogether, these results highlight the potential involvement of the AtCHX20 homologs for salt tolerance in grape but would require further characterization to validate their functions.”*

*We also added a sentence L402-403 to indicate the impact of the potential causative variants on gene function for the SNPs that overlap the second AtCHX20 homolog.*

*Lines 402-403: “Based on a snpEff analysis, these SNPs appeared to be mostly missense variants, causing codons that produce different amino acids.”*

*We also added a sentence L490-492 in the discussion section to comment on the putative impact of those variants on protein function.*

*Lines 490-492: “Interestingly, the SNPs were predicted to affect the amino acid composition of VITVgdSC2_v1.0.hap1.chr08.ver1.0.g144890 which could potentially have a significant impact on gene expression and protein activity [41].”*

Reviewer #2:

Cochetel et al. 2023 constructed a super-pangenome incorporating 9 north American wild grapevine genomes (haploid resolved) to investigate genomic variation across these individuals. They report intra- and intergenomic variation and test their super-genome by trying to detect variation previously reported for hybrids, sex determination, PD, and salt tolerance. Wild grapevines are an important resource of genetic diversity and understanding the genetic variation across genera is an important resource for grapevine improvement. Looking at wild grapevine genetic diversity at this scale is an advancement from previous wild grapevine studies. There are some questions and comments below that will help strengthen this manuscript:

General comments:

-Connect your supe-pangenome to use in other species for a broader impact of your work.

*Reply: We have added more details L431-433 to make sure the usefulness of our work for other species is emphasized.*

*Lines 431-433: “Our efforts provide an exemplar for future plant super-pangenomes and describe a pipeline that can be used in other species from the de novo assembly of diploid genomes to their pangenome construction.”*

*We also added a sentence L471-475 to highlight the great potential of such super-pangenome for other fruit crops.*

*Lines 471-475: “These results further support the broad potential and the flexibility of our pangenomics pipeline applicable in other species such as fruits crops (apples, pears, citrus, stone fruits trees, etc) by providing means to study the genetic variations between wild perennial accessions and the commercial hybrids derived from them."*

-Pangenomes are made up of multiple genomes of the same species to detect genetic diversity. Here, you only use one diploid genome (2 haploid) to construct the super-pangenome. I think a lot of intra-species variation is lost. The name "super-pangenome" is a little misleading, since pangenome implies multiple species' genomes are included. I think species-level pangenomes need to be assembled first, then they can be used to generate a genus (super) pangenome. This needs to be addressed in the manuscript. Otherwise, a different name might be more appropriate than "super-pangenome".

*Reply: A pangenome indeed represents multiple genomes of the same species. Based on literature, notably the review article by Khan et al. (Trends in Plant Science 25(2):148-158, 2020), a super-pangenome extends this definition by incorporating multiple species. A super-pangenome would include “at least one de novo assembly from each species”, feature that we provide in our super-pangenome. To prevent any confusion, we added a clear objective in the scope of our paper L90-91.*

*Lines 90-91: “Here, we describe a reference-unbiased super-pangenome of North American wild grapes in the genus Vitis with as a main objective to identify and analyze the interspecific genetic variability.”*

*In addition, several recent papers have been published using this nomenclature to differentiate species-level pangenome from multi-species pangenome with only one accession representing each species such as such as in watermelon (3 species used) or in tomato (11 species selected). Based on the current literature consensus, we believe that defining the pangenome we built as a super-pangenome stands correct.*

Comments by line:

Line 66- 86: You mention a single reference genome affects the intraspecific genetic variation, missing much of the genetic novelty within species, but then your super-pangenome only uses one genome per species.

*Reply: In the introduction section, we define the term pangenome and the motivations behind its creation. Further on, we mention the super-pangenome, with as main difference, the inclusion of more than one species and the representation of a genus instead of a single species. To avoid any dissonance, we added the main objective of the study in the sentence L90-91 to explain that our focus is the variability between species. While pangenomes were indeed primarily developed to prevent limitations of applications based on a single reference, our focus here was to use pangenomics tools to assess the genetic variability between species. Our work includes for now one accession of interest per species and could incorporate more in the future as we propose in the discussion L437-439.*

*Lines 437-439: “More accessions could also be added for the species already present in this super-pangenome to develop species-level pangenomes. We selected one accession per species to build the super-pangenome since intra-species variation was beyond the scope of this work.”*

Line 70: genetic landscape "within a species" that may…

*Reply: We integrated your suggestion L67-70.*

*Lines 67-70: “These limitations have motivated the development of pangenomes, which are transformative because they provide a comprehensive view of the genetic landscape within a species that may be otherwise overlooked [9].”*

Line 103: When you say "respective groups" do you mean species or are you talking about the 6 groups from the PCA analysis in Fig 1j? Please clarify why/how these were selected.

*Reply: We refer here to taxonomical groups defined previously where the species are separated based on their geographical distribution and phenological differences. We added this precision in the text L103-106.*

*Lines 103-106: “To construct a comprehensive representation of the North American Vitis genus, nine accessions were selected to be characteristic of their respective taxonomical group as defined previously based on their geographical distribution and phenological differences [3] and for their potential in breeding programs (Fig. 1a-j, Additional File 1: Table S1).”*

108: 791-6,925?

*Reply: For V. monticola (6,925), we attempted four times to produce a consensus optical genome map as mentioned in the materials and methods section L595-596 without success leading to the highest cumulated depth. However, since we did not use it, it was not indicated in the coverage range presented in the main results.*

*Lines 595-596: “Despite four next-generation mappings for V. monticola, we were not able to generate a consensus genome map for this genome.”*

*We also added a note regarding V. riparia in the materials and methods section L596-597.*

*Lines 596-597: “For V. riparia, no optical maps were available at the time of the assembly (Additional File 1: Table S3).”*

Line 110: Hybrid assemblies of both haplotypes?

*Reply: The word “diploid” was added to imply that the hybrid scaffolds contain the diploid information L111-113.*

*Lines 111-113: “The Falcon-Unzip contigs (N50 = 0.31-1.14 Mb; Table 1) were scaffolded using the genome maps to produce diploid hybrid assemblies (N50 = 1.13-9.22 Mb; Table 1).”*

Table 1: In the "Repeats" column, is this the % of the assembly that consisted of repetitive elements?

*Reply: Yes, grape genomes are known to be highly repetitive. The repeat percentages were extracted directly from repeatmasker report. The percentage was calculated to represent the non-overlapping number of bases of the assembly considered as a repetitive element. This clarification was added in the materials and methods section L620-621.*

*Lines 620-621: “For each assembly, the proportion of bases covered by repetitive elements were represented as repeat percentages.”*

Table 1 footnote: Delete the first sentence and just keep the second. It's not needed with he super script ones already in the table.

*Reply: Thank you for the suggestion, we corrected the footnote accordingly L122.*

*Line 122: “1 The values represent haplotype 1, haplotype 2, and unplaced sequences.”*

Line 123: Re-word. and "were used to constrict" a high-density Vitis…

*Reply: The sentence L124-126 was reformulated to present the approach first and then the results.*

*Lines 124-126: “The tool HaploSync [12] coupled with a high-density Vitis genetic map [13] was used to construct nine diploid sets of nineteen chromosomes with a target of at least 90% of anchored sequences and protein-coding gene loci (Table 1).”*

Line 131: These heterozygosity values for the wild grapevine genomes are high compared to what was previously reported. What was included in this calculation?

*Reply: For each genome, the assemblies of haplotype 1 and haplotype 2 were compared using nucmer as mentioned L607-609 of the materials and methods section. We have added more details to explain that the non-overlapping number of bases involved in any variant types were concatenated and used to represent the heterozygosity level compared to the assembly size of the haplotype used as reference L609-610 and L136-138.*

*Lines 607-610: “To evaluate heterozygosity, NUCmer from the tool MUMmer v.4.0.0beta5 [23] was used with the parameter “--mum” to align the haplotypes of each genome against each other. All the resulting variant types and lengths were concatenated together to estimate the proportion of the genome covered by variants (Additional File 1: Table S7).”*

*Lines 136-138: “To estimate the levels of heterozygosity in the nine Vitis genomes, we compared the assemblies of their phased haplotypes and considered every type of variants and any size.”*

*This comprehensive strategy is more inclusive than regular kmer or SNP-based approaches and uses assemblies as input instead of short reads whose mapping and kmer analysis are biased in structural variant sites resulting in important underestimation of heterozygosity in plants with high degree of structural variability like grapes. We have added values and references from previous studies with inclusive estimates as support L138-141.*

*Lines 138-141: “As expected, each genome presented high levels of variation between haplotypes, ranging from 16.79 to 23.77% of the genome sequence impacted by variants when the two corresponding haplotypes were compared (Additional File 1: Table S7) falling in the range of previous conservative estimates such as in Chardonnay (15.3%) [14] or Pinot Noir (11.2%) [15].”*

*We performed a complementary analysis L143-145 to provide estimates based on heterozygous SNP and found similar values than previous reports from literature.*

*Lines 143-145: “SNP was the most abundant type of variant with 1 SNP every 238-1159 bp (Additional File 2: Figure S1) as reported previously for wild grapes species [16,17].”*

*We obtained values in the same range from the graph decomposition L308-311.*

*Lines 312-315: “For each species, 1 heterozygous SNP was detected every 239-888 bp between haplotypes representing 0.11-0.42% of their assembly (Additional File 1: Table S17) which is consistent with our independent pairwise comparison using NUCmer and reports from literature [16,17].”*

Line 144: Fig S1 doesn't really add any new insights into the phylogenetic relationships compared to Fig. 1k, so it might not be necessary to include. Or you can include more of an explanation of why it is.

*Reply: We agree with the reviewer’s suggestion, the purpose of this figure was only to show the branches as amino acid substitutions per site. We removed Figure S1.*

144: What do you mean by "clock calibration"? Cite a source or include more of a description here.

*Reply: We reformulated the sentence L161 to use the term “molecular clock” instead of “clock calibration” and added a citation to a review which describes the different methods to estimate evolutionary rates including the one we used.*

*Line 161: “We also estimated divergence times at each node using molecular clock method [20] (Fig. 1k).”*

Line 145-147: Are the 450 rapidly evolving orthogroups specifically pictures in Fig. 1k? If so, highlight them/make them more apparent. If not, remove the figure citation.

*Reply: There are 450 rapidly evolving orthogroups in total. On Fig. 1k, the numbers represent at each node the number of orthogroups that show a significant expansion or contraction. For each node, the content of orthogroup detected in expansion or contraction can be different. We added some text in the legend to make it clearer.*

*“Positive and negative numbers indicated on the branches represent expansions and contractions, respectively, among the 450 rapidly evolving orthogroups as determined by gene family evolution analysis.”*

Line 154: delete "from"

*Reply: Thank you for the suggestion, we removed the word.*

*Lines 171-173: “After pairwise comparisons of assemblies between species, we inferred that 27.10 to 34.20% of the genome from each species was affected by genetic variants (Additional File 1: Table S11) [...]”*

Line 156: include the heterozygosity values again here in parentheses for the reader.

*Reply: The values were added L173.*

*Line 173: “[...] values that greatly exceeded heterozygosity (16.79-23.77%)”*

Line 167: You use the term "node" often, but never define what these are. Please define this the first time you reference it.

*Reply: A complete definition of the graph topology has been added L180-183.*

*Lines 180-183: “In the resulting graph topology, genomic sequences are represented as “nodes” that can be as small as one base pair. The chromosomes are stored as “paths” which are a linear representation of their DNA as traversals through the nodes.”*

Line 171: replace "species-specific" with "genome-specific" since you only have one representative genome per species.

*Reply: The adjective has been changed accordingly.*

*Line 191: “and iii) private genome; genome-specific nodes.”*

Lines 193-194: Clarify the meaning of this sentence, particularly "their effect on genetic components".

*Reply: This sentence L215-217 has been modified. We refer to the graph structure here.*

*Lines 215-217: “We incorporated these hybrid genomes to evaluate whether we could accurately assess their origins and to characterize their structural effects on the pangenome graph topology.”*

Line 202: Were the core and private genomes used the ones already determined by the super-pangenome or were these generated again for the three species and their hybrids compared here? Please clarify.

*Reply: We have modified the text to clarify L212-213. The genomes of the three species were added to the nine others to generate a new pangenome with 12 genomes.*

*Lines 212-213: “As expected, the addition of the three hybrids with the nine previous genomes [...]”*

Line 205: 57.2% of what? Also, I am not seeing this pictured in Fig S5a. In the text you say 57.2% were shared between the two haplotypes. This means about 43% (of the sequences?) were unique to the haplotype? That seems high.

*Reply: We simplified this entire analysis as described in L221-244, recalculated the shared node space, and now included it in the manuscript as a main figure as the reviewer recommended in one of the comments above. We decided to only represent the nodes that are shared between the haplotypes compared as a measure of their similarity. We also added a more detailed description of the node space shared as barplot or boxplots to make it easier to understand for the reader.*

*Lines 221-244: “To evaluate genome relatedness, we focused on the three hybrids and the three parental species from which the hybrids were derived. We quantified the degree of relationship between the different genomes using the total length of the nodes they shared as a metric (Fig. 3). Three levels of comparisons were investigated: intra-genome by the comparison of the two haplotypes within each genome (Fig. 3a-c), inter-haplotype by the comparison of the hybrids’ haplotypes to their parents (Fig. 3d-f), and inter-genome comparing haplotypes of different species (Fig. 3f-i). First, intra-genomic comparisons were performed between the two haplotypes of each genome; on average, 60.2% was shared between the two haplotype sequences of each species (Fig. 3b-c). Notably, the parents presented a higher percentage of shared node space between their haplotypes (65.9% on average) compared with the hybrids (54.4% on average). The hybrids having V. berlandieri as a parental species showed the lowest percentage (Richter 110R: 51.7%; Kobber 5BB: 50.9%), probably because this species is genetically distant from the two other parents V. riparia and V. rupestris (Fig. 1k). Secondly, we evaluated if the pangenome could detect the relatedness of the hybrids with their parental species. Each hybrid haplotype was derived from a different species, as determined during their assembly [7]. When we compared each hybrid haplotype (e.g. 110R haplotype berlandieri annotated 110R be on Fig. 3d) with the haplotypes of its parental genome (i.e. V. berlandieri haplotype 1 or 2 annotated be hap1 or hap2 on Fig. 3d), we found on average 58.2% of their nodes being shared. V. rupestris showed the most conserved node space with its corresponding hybrids 110R and 101-14 Mgt (Fig. 3e-f). Lastly, the smallest percentages of shared node space were detected when the genomes were compared in an inter-specific configuration (Fig. 3g-i). As expected, V. riparia and V. rupestris haplotypes showed the highest shared node space since there are closer to each other than V. berlandieri. Despite those similarities, the overall shared node content between different species remained lower than the percentages observed when hybrid haplotypes were compared with their parental species.”*

*In addition, we restated clearly in L215-219 that the aim of this analysis is to mostly support the pangenome graph structure.*

*Lines 215-219: “We incorporated these hybrid genomes to evaluate whether we could accurately assess their origins and to characterize their structural effects on the pangenome graph topology. We established different types of comparisons to assess whether the pangenome graph was able to reflect correctly different degrees of similarity between close or distant species.”*

Line 212: Replace "in" with "on".

*Reply: We replaced “in average” occurences by “on average” throughout the manuscript.*

Line 213: What % of the hybrid genomes was the parental genomes able to explain. All of it?

*Reply: Our aim here was mainly to make a relative assessment of the accuracy of the pangenome to capture intra-genomic, hybrid-parents, and inter-genomic relationships, which was the case. Overall, we found 58.2% of the node space shared between the hybrid genomes and their parents but additional analyses would be necessary to assess how much parental genome can explain hybrids. Here, the nodes come from a graph with more species than the ones involved in the hybrid-parents trio which is why we can only provide a relative assessment of the different comparisons we performed.*

Line 220: Significant time and space is spent on this hybrid comparison, so would be better to have a main figure to illustrate your findings.

*Reply: We agreed with this suggestion and added our re-analysis as the main figure 3.*

Line 229: Discarded for or excluded from? Were they set aside to study specifically?

*Reply: Thank you for the suggestion, the sentence was reformulated L255-257. We excluded them from any further analyses as their structure does not allow us to class them unambiguously. We provide a more detailed answer in response to your next comment.*

*Lines 255-257: “The genes not falling in any of the above categories were classified as ambiguous; these represented on average only 2.6% of the gene content and were excluded from subsequent analyses (Additional File 1: Table S12).”*

Line 229: What did you find related to the other categories. It seems like this is what you set out to find, but you don't report on it in the text or main figures. The % of core, dispensable, and private genes? The reader expects a discussion on this, but this comes later. You can re-word or restructure this paragraph so it has more of a logical flow.

*Reply: About 2% of the genes were classified as ambiguous because they had a complex node composition, i.e. their sequence being represented by a mix of nodes belonging to the core, dispensable, and private classes (e.g., 50% core and 50% private). A non-ambiguous classification of those genes was not possible, so we decided to discard them. We believe that the 98% of the gene content selected for our analyses is representative of the gene space.*

Lines 247-249: If the genes arose through duplication, TEs could also be a mechanism for origination.

*Reply: We elaborated our discussion about TEs to include this interesting aspect L446-449.*

*Lines 446-449: “TEs can also influence the gene content itself (Fig. 4i) by numerous mechanisms such as gene mutation, gene movement, but also duplication via unequal crossing over [35,36], which could all significantly contribute to the control of the variable genome size of a genus.”*

Line 254: Do you mean genes in these rapidly evolving gene families? Fig 3K looks to show % of genes.

*Reply: For each genome, we classified the genes that belong to the rapidly evolving gene families identified using CAFE into the different pangenome classes (core, dispensable, and private) and calculated the respective proportions. We reformulated the sentence L282-284 to make the point clear.*

*Lines 282-284: “Among the genes belonging to the rapidly evolving families identified during the phylogeny analysis, the proportion classified as variable genes (dispensable/private) was twice as high (Fig. 4k) as the core genes (Kruskal-Wallis, P value < 0.05).”*

Line 261: This is interesting, since, at this level of GO, it looks like some of these terms are related to basic functions, like embryo development. Maybe some of these variable genes evolved important/essential functions in their species/individual.

*Reply: We agree with your comment and commented L289-291.*

*Lines 289-291: “The enrichment of basic functions for the variable genes such as embryo development does not rule out the evolution of important functions for specific species.”*

Line 267: I don't think you can make this conclusion without functional tests. Maybe a more general statement can be made.

*Reply: We agree with the reviewer. We removed the wording implying disposable and toned down the sentence L296-300 to generate a more general statement.*

*Lines 296-300: “The pool of private and dispensable genes does not seem to be conserved, at least under certain environmental conditions, suggesting that they might provide benefits for local adaptation to specific abiotic and/or biotic stresses and could be responsible for significant phenotypic variation among species.”*

*Line 277: delete "across"*

*Reply: Thank you for the suggestion, the word “across” was deleted.*

*Lines 307: “the pangenome and NUCmer-based inferences were consistent [...]”*

Line 278: change 86 to 76%? Check table S16.

*Reply: The suggestion is correct, we corrected the number L307-308.*

*Lines 307-308: “[...] presenting 90% and 76% of SNPs and INDELs, respectively (Additional File 1: Table S16)”*

Line 281: re-word

*Reply: Thank you for your suggestion, we reformulated the sentence L309-312.*

*Lines 309-312: “By classifying the variants in different groups of sizes, we observed that more than 96% of the total were short-length variants with 1-bp variants (most SNPs) as the most abundant size and the remaining set being shorter than 10 bp (Fig. 5b).”*

Line 281: delete "most"

*Reply: Thank you for your suggestion, we reformulated the sentence as mentioned above.*

Lines 286-287: Put "except for the multiple nucleotide polymorphisms (MNP)" outside of the parentheses and then site S7a.

*Reply: We removed the variant types MNP and other from our analysis since they mostly come from complex variations structures from the graph decomposition as discussed L776-778 of the materials and methods section.*

*Lines 776-778: “While the variant types MNP (Multiple Nucleotide Polymorphism) and other were also detected in the vcf file, they were not considered further as they correspond to complex variation scenarios from the decomposition of the graph into variants.”*

Line 294: Is this because SV was not able to be detected outside of the haplotypes of the species? What is a modifier site?

*Reply: We added an explanation L326-330. A private gene is composed of at least 80% of nodes only found in a specific genome. Only non-private nodes, if any, within the private gene sequence would allow the formation of a variation structure within the pangenome graph.*

Lines 326-330: “The private genome was particularly less impacted by variants when compared with the core or dispensable genomes confirming that the sequence of a private gene is mostly genome-specific and do not share many nodes (considered here as potential variant sites), if any, with the other genomes.”

*Reply: We added a brief description for the modifier sites being variants mostly present in non-coding regions L330.*

*Line 330: “Regarding the modifier sites (usually variants in non-coding regions) [...]”*

Line 298: It's worth to briefly mention how this gene contributes to sex determination.

*Reply: A sentence has been added regarding the potential role of this gene in sex determination L348-351.*

*Lines 348-351: “We focused on the variants impacting VviINP1, known to be involved in pollen aperture formation [6]. In the female allele, the sequence of this gene contains an 8-bp deletion which has been suggested to be responsible to the non-functionality of the corresponding protein [6].”*

Line 302: Any new or genome-specific variation of interest in the SDR discovered via the super-pangenome?

*Reply: Thanks for this suggestion. We have performed new analyses of the pangenome focused on the SDR presented on Fig. 5f. We localized in all the genomes the boundaries of the SDR. We extracted the corresponding nodes from the graph and identified allele-specific nodes (female or male). We studied their distribution along the SDR and observed an accumulation of allele-specific nodes in positions of the SDR known to presence a strong association to the sex allele. We then examined the repeat distribution and observed an accumulation in regions specific to the male allele. We also added a snpEff analysis using the allele-specific variants and confirmed that the graph captures the INDEL in VviINP1.*

*Lines 339-356: “To validate the variants derived from the pangenome graph topology, we investigated its structure at the grape sex-determining region (SDR) (Fig. 5f). For both alleles (female and male), we observed a striking allele-specific accumulation between the TREHALOSE-6-PHOSPHATE-PHOSPHATASE (TPP) and INAPERTURE POLLEN 1 (VviINP1) genes which correspond to the peak of linkage disequilibrium previously observed in this region [6]. In the females, allele-specific nodes were also accumulated around the Flavin-containing monooxygenase (FMO) loci which might be involved in the conservation of their function as they are not all protein-coding in the males [6]. The density of allele-specific nodes was also noticeably overlapping the repeats distribution suggesting an important role of repetitive elements in the larger size of the male allele. Few SNPs and INDELs were found to be conserved in an allele-specific manner. We focused on the variants impacting VviINP1, known to be involved in pollen aperture formation [6]. In the female allele, the sequence of this gene contains an 8-bp deletion which has been suggested to be responsible to the non-functionality of the corresponding protein [6]. Remarkably, the super-pangenome graph captured the known variants distinguishing the INP1 male and the female alleles (Fig. 5f), including an allele-specific SNP at 60 bp from the VviINP1 transcriptional start site (TSS) and the 8-bp INDEL starting at 74 bp from the TSS (Additional File 2: Figure S7). Moreover, the flower sexes of all the accessions used to build the pangenome were predicted correctly by their alleles in the pangenome and confirmed with phenotyping data from the field (Fig. 1a-i).”*

*We also added discussion elements regarding the repeats where we elaborate about repetitive elements L449-453.*

*Lines 449-453: “Interestingly, the accurate representation of the sex-determining region in the pangenome graph evidenced an allele-specific pattern for the repeat distribution as previously reported in grapes [37]. The allele-specific nodes confirmed an accumulation of repetitive elements in the male allele, mechanism involved in the evolution of heteromorphic sex chromosomes [38].”*

Lines 304-311: You found SNP variants at these regions, and you would expect to see this, since V. arizonica is included and had those SNPs. Were you just verifying if you could pick up on these SNPs in the pan genome or were you trying to see if you could predict PD resistance of other species included in the analysis by determining if they also had them? Are these known conserved genes involved PD across species? Do you know the PD resistant phenotype of the other species? Can you take this analysis a step further and make predictions? Clarifying the aims of this analysis will make it clearer for the reader.

*Reply: We agree and explained it L358-363 to make it clearer. We were trying to see if we could detect polymorphisms at the same SNP locations as described in recent work (Morales-Cruz A, Aguirre-Liguori JA, Zhou Y, Minio A, Riaz S, Walker AM, et al. Introgression among North American wild grapes (Vitis) fuels biotic and abiotic adaptation. Genome Biol. 2021;22:254.) since not all the genomes included in the pangenome are PD resistant. Our aim was not to predict PD resistance per se, which would require more phenotype data and a larger panel, but validate the presence of known polymorphisms associated with PD resistance in the pangenome graph.*

*Lines 358-363: “To further explore the variants embedded in the pangenome graph and gather information over multiple chromosomes, we also investigated polymorphisms associated with Pierce’s disease resistance. Eight peaks of association were previously detected in V. arizonica [25] (one of the input genomes integrated into the pangenome) using genome-wide association studies (GWAS). As the other genomes included in the pangenome were not selected for Pierce’s disease resistance, we expect to detect the polymorphisms.”*

Line 312: Mapping the reads or do you mean identifying SNPs/variation from the last GWAS?

*Reply: The idea is that the reads that were mapped from the last GWAS can be mapped on the pangenome, and the same polymorphisms will be detected without any variant calling since they are already embedded as nodes in the graph. We reformulated the sentence L366-370.*

*Lines 366-370: “In other words, almost the entirety of the regions previously detected de novo by variant calling was represented in the pangenome graph structure. As a result, mapping the reads from the previous GWAS on the pangenome should in theory only require genotyping the embedded variants to detect the associated polymorphisms, thereby bypassing any variant calling.”*

Line 355: Can you determine if it is an expression difference or maybe a gene dosage difference contributing to the salt tolerance?

*Reply: Unfortunately, we do not have expression data from the accessions used for the GWAS, nor have enough replicates to compare the expression between the genomes included in the paper based on salt tolerance. Regarding gene dosage, we did not observe any gene content difference in this region between the salt excluder V. girdiana SC2 and the salt accumulator V. arizonica B40-14 (Fig. 6e).*

Line 386: And can influence gene duplication via unequal crossing over. You can elaborate a little more about TEs in the discussion and role in genome evolution.

*Reply: Gene duplication is an interesting potential mechanism that could be behind the control of the variable genome size. We included it as suggested L446-448 and elaborated more about TEs.*

*Lines 446-448: “TEs can also influence the gene content itself (Fig. 4i) by numerous mechanisms such as gene mutation, gene movement, but also duplication via unequal crossing over [35,36] [...]”*

Lines 392-393: Why?

*Reply: We have added more content and citation to support it L460-464.*

*Lines 460-464: “First, the dispensable genome increases with the addition of more samples (Fig 2a, 3a), and this trend is likely to continue based on the slopes predicted from our modeling until reaching a plateau for the gene content and most likely further increasing for the sequences as suggested for eukaryotic genomes [39].”*

Lines 417-419: This should be mentioned in the results section and the discussion of the role of these genes in other species can be discussed here.

*Reply: We moved the discussion part regarding the roots gwas in the results L409-412 and transferred the discussion about the role of the genes in other species in the discussion section L492-497.*

Lines 426-441: This is somewhat repetitive with the discussion.

*Reply: The entire conclusions were re-written to be more concise regarding our results and provide more perspectives.*

*Lines 508-523: “In summary, we constructed a super-pangenome to represent and analyze North American wild species of the Vitis genus. The assembly of phased diploid genomes for the nine selected species was a fundamental starting point to ensure an accurate representation of genetic variations that occur within and between genomes. We were able to navigate through different layers of information integrated within the pangenome graph from large structural variations to single nucleotide polymorphisms. We investigated and interpreted the genomic diversity within the genus and provide novel insights regarding the core and the variable genome in grapes. We expect that many more insights on Vitis evolution as the pangenome for this genus grows and integrates more species, notably from distinct geographical regions such as Europe and Asia, which should help to further elucidate grapevine domestication for example. The integration of the V. vinifera species will provide direct insights into the genetic bases of the organoleptic properties of the wine-producing berries [45]. The concept of pangenomics provides many opportunities to finally move from a single-reference to a reference-free approach that incorporates broad genetic diversity when comparing different genomes. Significant improvements in accuracy are expected for approaches sensitive to single nucleotide changes such as transcriptomics and genetic association studies of populations with frequent inter-species introgressions.”*

Line 445: List the tissues or say (see corresponding sections below).

*Reply: We have added “(see corresponding sections below)” L527.*

*Line 527: “Several plant tissues (see corresponding sections below) were collected for sequencing of DNA [...]”*

Line 448: maintained in the field or greenhouse

*Reply: We have added “in the vineyards” L530.*

*Line 530: “Most of the samples were collected from accessions maintained in the vineyards [...]”*

Line 448: Was there a specific reason you selected the genera you did? Availability of genome sequences, *plant material, or phylogenetic relationships? State this here.*

*Reply: We added a sentence L532-533.*

*Lines 532-533: “The selection of the different accessions was based on plant material availability and agronomical interests evidenced previously.”*

Line 462: Delete "a"

*Reply: It was deleted.*

Line 503: Specify if the sequences were assembled into consensus contigs or haplotype-resolved.

*Reply: We specified the type of contig and type of assembler L587-588.*

*Lines 587-588: “For each genome, SMRT sequences (Pacific Biosciences, CA, USA) were assembled into haplotype-resolved contigs using the diploid-aware assembler Falcon-Unzip [46] [...]”*

Line 515: BUSCO scores

*Reply: We have added “scores” after the tool version L600-601.*

*Line 600-601: “Gene space completeness was evaluated using BUSCO v.3.0.2 scores [50].”*

Line 523: Align haplotype of each genome against each other or against the primary haplotype?

*Reply: Per genome, haplotype 1 is compared to haplotype 2 in vice versa, we reformulated the sentence L607-609 to make it clearer.*

*Lines 607-609: “To evaluate heterozygosity, NUCmer from the tool MUMmer v.4.0.0beta5 [23] was used with the parameter “--mum” to align the haplotypes of each genome against each other.”*

Lines 523-524: Variants of any size? More detail is needed here, specially since your heterozygosity scores are higher than past reports for wild grapevines.

*Reply: We have added more details as mentioned previously L609-610. All the variants of any size or any type were concatenated together to estimate the levels of heterozygosity.*

*Lines 609-610: “All the resulting variant types and lengths were concatenated together to estimate the proportion of the genome covered by variants (Additional File 1: Table S7).”*

Lines 591-595: Again, please define what a node id and sequence are comprised of. Are these sequences of a defined length, coding and/or non-coding, single nucleotides?

*Reply: Now the manuscript contains a definition of those terms L180-183 as suggested previously.*

*Lines 180-183: “In the resulting graph topology, genomic sequences are represented as “nodes” that can be as small as one base pair. The chromosomes are stored as “paths” which are a linear representation of their DNA as traversals through the nodes.”*

Line 614: define "decoys"

*Reply: We added a definition for the decoy regions L707-709.*

*Lines 707-709: “Decoy regions from the genome are used during the scoring of the alignments to reduce false mapping of sequenced fragments that originate from unannotated genomic loci [80].”*

Main Figures:

Fig 1- include the name of the species in panels a-i or somehow connect species names in j and K with the species in panels a-i. Also, these species are dioecious, right? Indicate if the flowers/individuals pictures are male or female.

*Reply: The names of each species and the sex of their flowers were added on the panels a-i.*

Fig 3g: The bar graph looks off. Are the white boxes supposed to show the % of the genes without annotated domains? It's better to make the outlines of the bars black if that is the case.

*Reply: The graph was modified accordingly.*

Fig 3k- change Y axis label to % genes in expanded/contacted gene families

*Reply: The graph was modified accordingly.*

Fig 3l, m: would be helpful to add light lines from the category to the bubbles, especially for the core genes, so it is easier to see which one corresponds to which.

*Reply: The graph was modified accordingly.*

Fig 4a- define abbreviations in legend.

*Reply: The abbreviations are now defined in the legend.*

Fig 4d- The Y-axis descriptions are not clear from the figure or the legend. Why is intron listed in the top row and bottom row? Are they referring to different types of mutations in the intron? Why is "disruptive" and "conservative inframe" listed with "insertion" and "deletion" after it, when those are headings of the columns? Is "high", "moderate" and "low" referring to synonymous, missense, and stop codons or to the impact of all types of mutations based on location within the gene? Please clarify.

*Reply: The results from Fig. 4d were re-analyzed and are now presented on Fig. 5c. We made it simpler and summarized the different effects for the same impact. On the previous version, there was indeed the same effect in multiple impacts because the combination of multiple effects can trigger a single effect to produce different impacts.*

Figure 4f: Fig 4f is difficult to interpret given the current legend. It is not clear what the dots and lines connecting them mean. Why are the 8 peaks separated as they are in the vertical bar graph. Does “set *size” mean number of genomes that had SNPs? Please clarify.*

*Reply: A more detailed explanation was added to the legend to clarify the figure which is now Fig. S8.*

Fig 5a- Are there supposed to be lines connecting the boxes? It looks like there is a lot of white space in this panel.

*Reply: Fig. 5a is now Fig. 6a. It was updated to contain a description of each step and be self-explanatory. The requirements of each step regarding inputs from previous steps can now be inferred from the figure.*

Supplements:

Sup Table 1- Write out FPS abbreviation

*Reply: The abbreviation was spelled out.*

Sup Table 2- Why is the N50 for B40-14 so low compared to the others? Check this value.

*Reply: We checked the value, and it is correct. Generating nine genome assemblies took a significant amount of time including assembly and annotation optimizations. V. arizonica was the first Vitis genome of this project to be sequenced and the sequencing platform was also in active optimization. The statistics (N50) are similar to published genomes (Muscadinia rotundifolia) that were sequenced at the same time.*

Sup Table S3- Why does V. riparia not have values in this table. Include a note under the table or in the M&M to explain.

*Reply: A note was added for V. riparia L596-597 following the explanation for V. monticola.*

*Lines 596-597: “For V. riparia, no optical maps were available at the time of the assembly (Additional File 1: Table S3).”*

Sup Table 4- Why no Iso-seq for V. rupestris?

*Reply: A note was added L626-628.*

*Lines 626-628: “For V. rupestris, IsoSeq reads were not available at the time of the annotation, RNA-Seq was used as transcriptomic evidence (Additional File 1: Table S4).”*

Sup Table 9- define SD. Did 9031, HP-1 and B38 not have genome size predictions?

*Reply: SD is now defined. The values for V. berlandieri, V. riparia, and V. rupestris were retrieved from literature to complete the table as we do not have flow cytometry estimates for those accessions. We added a sentence L659-660.*

*Lines 659-660: “For V. berlandieri, V. riparia, and V. rupestris, estimates from the same species were retrieved from literature [77].”*

Sup Table 14- define/rename "number" and "total" so it is more descriptive. Is one for graph inferred and the other orthology?

*Reply: The column names were renamed to be more descriptive. The number of consistent genes correspond to genes found in the same class in both the graph-inferred and the orthology approach. When this number is compared to the total number of genes in the class, we calculate the percentage.*

Sup Table 16- What is length and coverage referring to? The title says consistence statistics between nucmer and vg deconstruct but that comparison is not clear from the headers.

*Reply: The column names were renamed to be more descriptive.*

Fig S5a and b: In S5a, if this is an intragenomic comparison, then why is the X-axis the pangenome length? Does incorporate all the data for the 3 species? Isn't this showing what percentage of the haplotypes that consisted of core and private sequences? There is a disconnect between what is discussed in the manuscript and what is shown in Fig S5. A more descriptive legend or a more detailed description would help connect the figure to the manuscript text. In S5b, label your samples better and/or tell what the IDs mean in the legend. Also, I don't see an "outline color" that is supposed to denote the genome class. What do the yellow, orange, red, purple, blue, and teal colors mean? Do they specify a specific genome? *This needs a key.*

*Reply: Fig. 3 now replaces the former Fig S5. We hope the new details added to it makes it clearer and answer your questions.*

Sup Tables in general- Mention why data points are missing in supplemental tables for specific genomes.

*Reply: We went through all the tables as recommended above and made sure there is an explanation when needed.*

**Second round of review**

**Reviewer 1**

In the revised manuscript, the authors have addressed most of my concerns. I still have several concerns.

1. The title "A super-pangenome of the North American wild grape species (Vitis spp.) reveals genus-wide association with salt tolerance" is not suitable for the maintext content. In my opinion, the major contributions to the community in this MS: (1) Haplotype-resolved genome assemblies of the representative accession of nine species in Vitis genus. (2) Identification and characterization of the comprehensive genomic variants between two haplotypes of a single species and across nine species. (3) Some evolutionary and trait related insights. The content related to the candidate gene conferring salt tolerance only accounts for a small fraction of this MS, and thus it should not be emphasized in the title. In addition, the authors insist that a super-pangenome could be used here. I suggest that "haplotype-resolved genome assemblies of nine North American wild grape species" is more appropriate.

2. Line 161-163: "we aligned in a pairwise configuration the nine haplotype 1 proteomes". For each species, the haplotype 1 and 2 are assigned randomly, and thus the assigned haplotype 1 of different species may be not the corresponding one. It's better to clarify these for the kind of analyses.

3. Pan-GWAS was conducted to detect significantly associated variants with salt tolerance using 153 samples from 12 species. I did not notice the exact number of samples for each species. On chromosome 8, a significant peak was observed. To provide more evidence for this gene, the authors can analyze these samples extensively. For each species, what about the relationships between the chloride concentration and the variants?

**Reviewer 2**

The authors adequately addressed my questions in their revised manuscript and responses.

**Authors Response**

**Point-by-point responses to the reviewers’ comments:**

Reviewer #1:

In the revised manuscript, the authors have addressed most of my concerns. I still have several concerns.

1. The title "A super-pangenome of the North American wild grape species (Vitis spp.) reveals genus-wide association with salt tolerance" is not suitable for the maintext content. In my opinion, the major contributions to the community in this MS: (1) Haplotype-resolved genome assemblies of the representative accession of nine species in Vitis genus. (2) Identification and characterization of the comprehensive genomic variants between two haplotypes of a single species and across nine species. (3) Some evolutionary and trait related insights. The content related to the candidate gene conferring salt tolerance only accounts for a small fraction of this MS, and thus it should not be emphasized in the title. In addition, the authors insist that a super-pangenome could be used here. I suggest that "haplotype-resolved genome assemblies of nine North American wild grape species" is more appropriate.

*Reply: We followed the Editor’s recommendation and changed the title to: “A super-pangenome of the North American wild grape species”.*

2. Line 161-163: "we aligned in a pairwise configuration the nine haplotype 1 proteomes". For each species, the haplotype 1 and 2 are assigned randomly, and thus the assigned haplotype 1 of different species may be not the corresponding one. It's better to clarify these for the kind of analyses.

*Reply: To identify single copy orthologs, we used Orthofinder which requires as input the protein representation of a single allele for each gene. We decided to use the haplotype 1 for each species as it presented systematically the highest BUSCO score of the two haplotypes in all the species and was generally the longest haplotype with the most genes. We added a sentence L162-164 to clarify.*

*L162-164: “Orthogroups were identified using the haplotype 1 as representative as it presented systematically the highest BUSCO score of the two haplotypes and was generally the longest haplotype with the most genes.”*

3. Pan-GWAS was conducted to detect significantly associated variants with salt tolerance using 153 samples from 12 species. I did not notice the exact number of samples for each species. On chromosome 8, a significant peak was observed. To provide more evidence for this gene, the authors can analyze these samples extensively. For each species, what about the relationships between the chloride concentration and the variants?

*Reply: For each sample, the species is indicated in Table S17. Although many samples correspond to the species V. arizonica, as it was the focus of previous studies, accessions were collected to represent a broad range of salt tolerance, and the selection was independent of the species. However, the sampling distribution does not provide us with sufficient power and resolution to work at the species level and to make inferences from it. Furthermore, as we limit our conclusions to identifying these genes as candidates for salt tolerance, we believe that further analysis is beyond the scope of the current work. As suggested by the reviewer in the previous review, in the discussion, we stress the hypothetical role of the two candidate genes based on their association in panGWAS and outline further experiments that should be conducted to test the functions of these candidate genes in salt tolerance.*
